# Supplementary material for: Metagenomic of Liver Tissue Identified at Least Two Genera of Totivirus-like Viruses in Molossus molossus Bats
Source: Microorganisms. 2024 Jan 19;12(1):206. doi: 10.3390/microorganisms12010206 (PMC10819564; doi:10.3390/microorganisms12010206)
Supplement: Supplementary file 1 [file microorganisms-12-00206-s001.zip › microorganisms-2765255-supplementary.pdf]

SUPPLEMENTARY MATERIAL:

Metagenomic of liver tissue identified at least two genera of totivirus-like in *Molossus molossus* bats

Roseane da Silva Couto[1,†], Endrya do Socorro Foro Ramos[1,†],Wandercleyson Uchôa Abreu [2,†], Luis Reginaldo Ribeiro Rodrigues [3], Luis Fernando Marinho [4], Vanessa dos Santos Morais [5], Fabiola Villanova [1], Ramendra Pati Pandey [6], Xutao Deng [7], Eric Delwart [8], Antonio Charlys da Costa [5,‡] and Elcio Leal [1,*,‡]

[1] Laboratório de Diversidade Viral, Instituto de Ciências Biológicas, Universidade Federal do Pará, Belem 66075-000, Pará, Brazil; couto.roseane@gmail.com (R.S.C.); endrya.ramos@gmail.com (E.d.S.F.R.); fvillanova@gmail.com (F.V.)

2 Programa de Pos-Graduação REDE Bionorte, Polo Pará, Universidade Federal do Oeste do Pará, Santarém 68040-255, Pará, Brazil; uchoa_vet@yahoo.com.br (W.U.A.).

3 Laboratory of Genetics & Biodiversity, Institute of Educational Sciences, Universidade Federal do Oeste do Pará, Santarém 68040-255, Pará, Brazil; luisreginaldo.ufpa@hotmail.com (L.R.R.R.).

4 University of Amazonia, Santarém 68040-255, Pará, Brazil; fm8885785@gmail.com (L.F.M.).

5 Laboratory of Virology (LIM 52), Instituto de Medicina Tropical, Universidade de São Paulo, São Paulo 05403-000, Brazil; va.morais@usp.br (V.d.S.M.); charlysbr@yahoo.com.br (A.C.d.C.)

6 School of Health Sciences & Technology UPES University, Dehradun, Uttarakhand, India Campus Energy Acres, PO Bidholi, Dehradun-248007; ramendra.pandey@gmail.com (R.P.P.).

7Vitalant Research Institute, San Francisco, CA 94143, USA; edelwart@vitalant.org (E.D.) xutaodeng@gmail.com (X.D.)

8 Department Laboratory Medicine, University of California San Francisco, San Francisco, CA 94143, USA

*Correspondence: elcioleal@gmail.com (E.L.)

†These authors contributed equally to this work.

‡ These authors jointly supervised this work.

FIGURES:

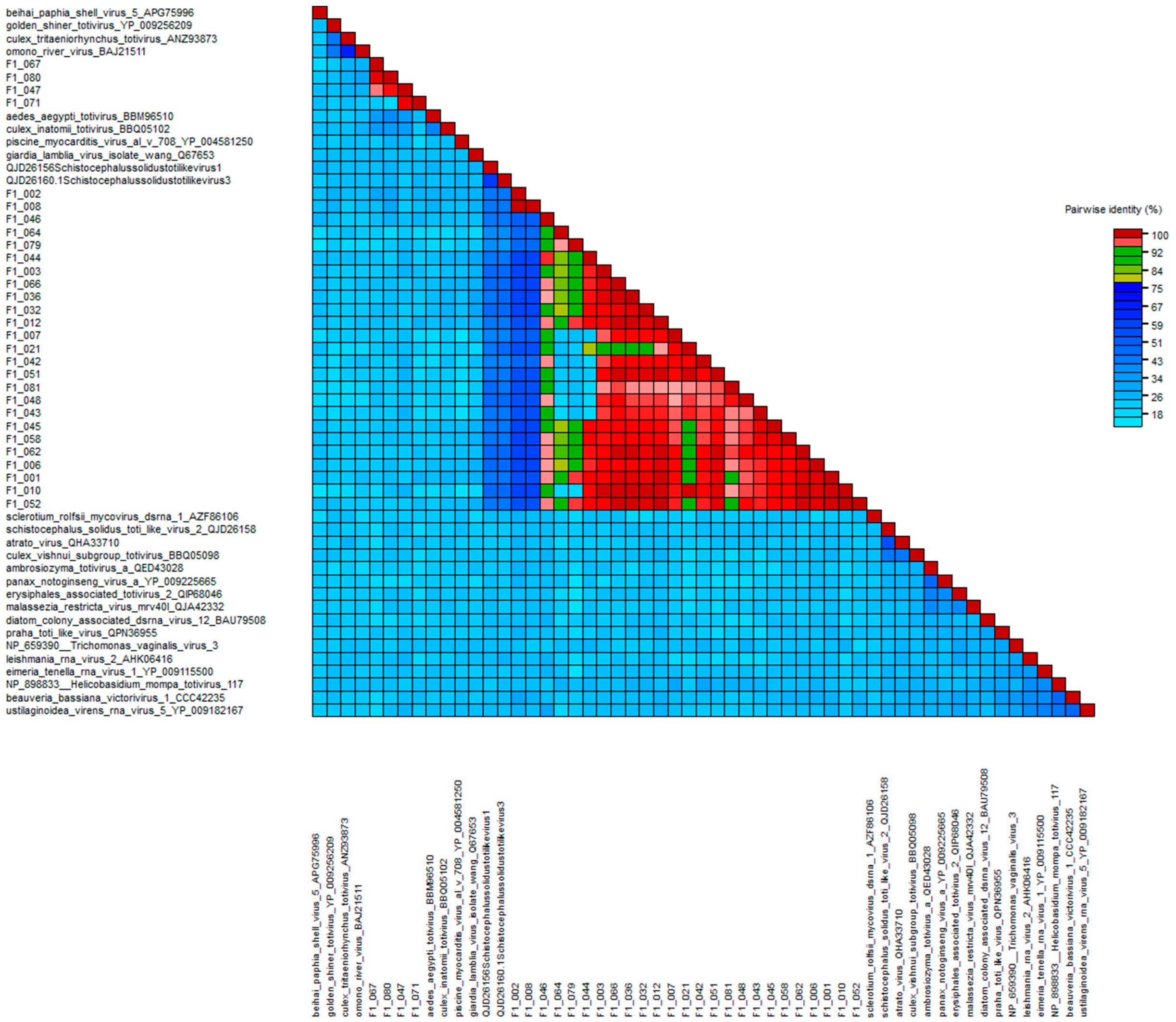

Figure S1: Amino acid dentity of RdRpol of totiviruses. Pairwise identity of sequences generated in this study and some reference sequences of totiviruses. The colored scale indicates the percentage of identity.
